# Supplementary figures and images for: Constriction imposed by basement membrane regulates developmental cell migration
Source: PLoS Biol. 2023 Jun 28;21(6):e3002172. doi: 10.1371/journal.pbio.3002172 (PMC10335704; doi:10.1371/journal.pbio.3002172)

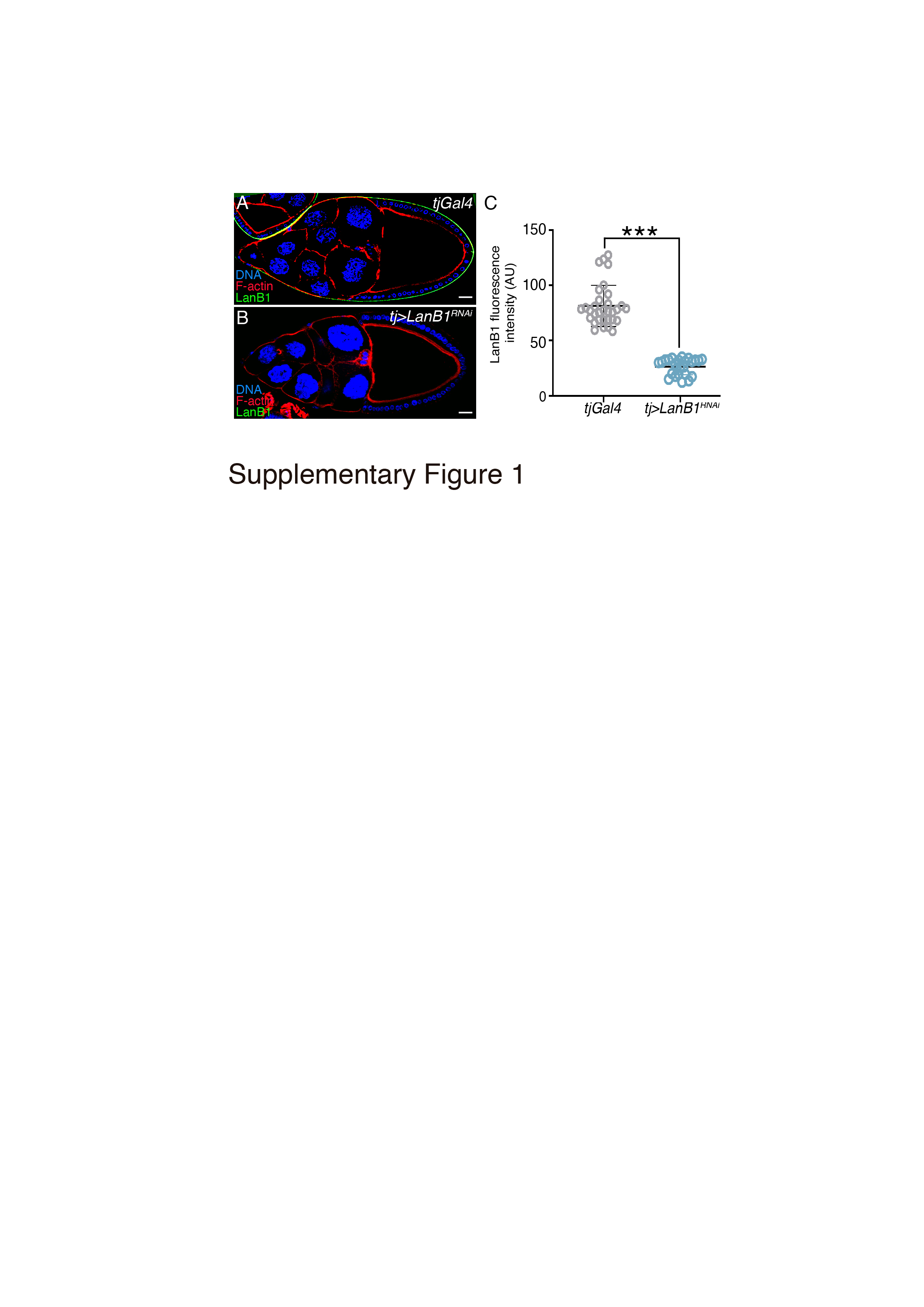

Supplement: S1 Fig — (A) S10 control tjGal4 and (B) tj>LanB1RNAi (B) egg chambers stained with anti-LanB1 antibody (green), the DNA marker Hoechst (blue) and the F-actin marker Rhodamine-Phalloidin (F-actin, red). (C) Quantification of the LanB1 levels in egg chambers of the specified genotypes. The statistical significance of differences was assessed with a t test, *** P value < 0.001. Horizontal and vertical lines indicate mean and SD, respectively. Scale bars in A and B, 20 μm. The raw data underlying panel C are available in S1 Data. (JPG) [file pbio.3002172.s015.jpg]

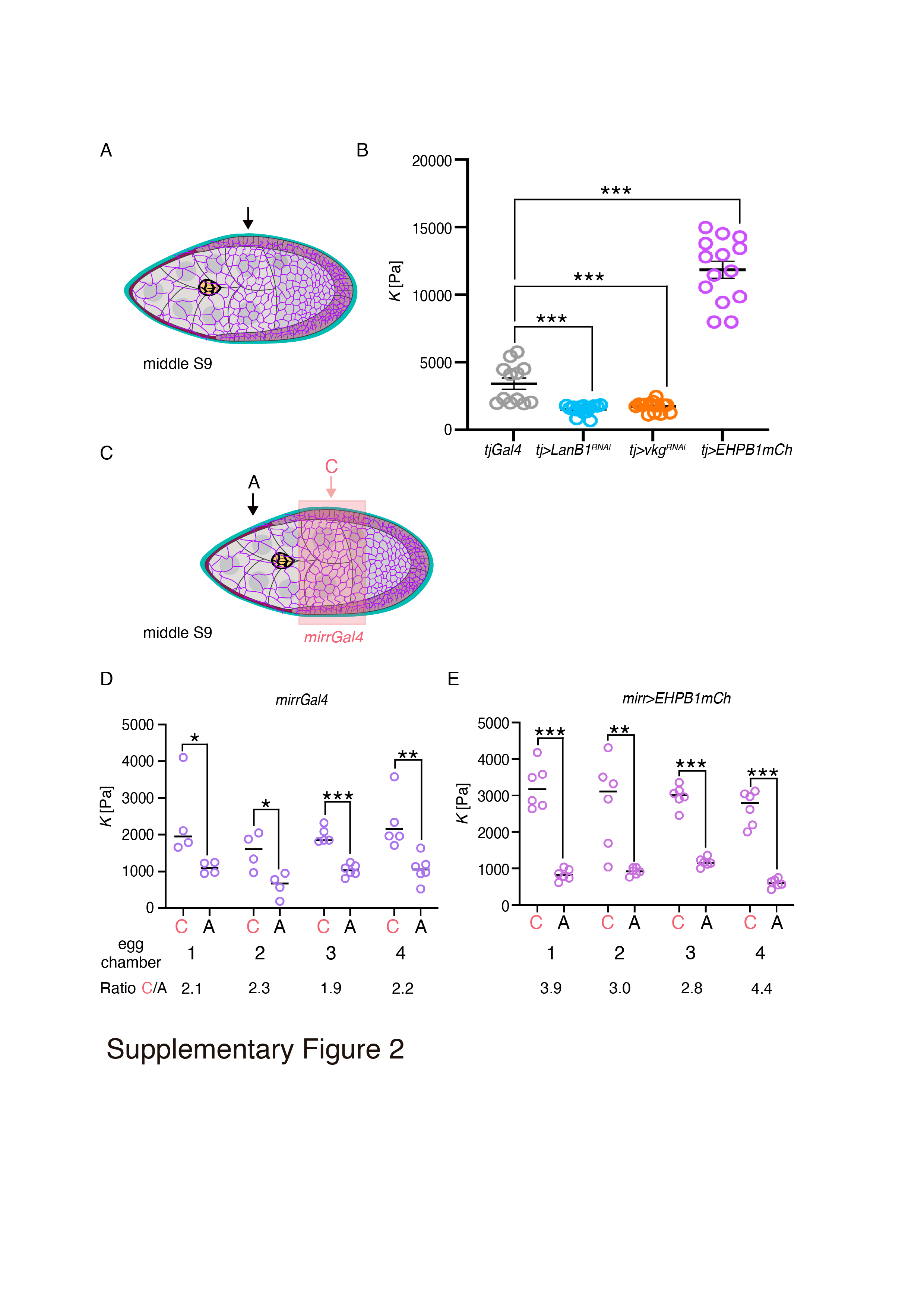

Supplement: S2 Fig — (A) Schematic drawing of an early S9 egg chamber illustrating the BCs (yellow), NCs (gray), FCs (purple), BM (green), and the position where AFM measurements were taken (arrow). (B) Comparison of the apparent elastic modulus K in egg chambers of the designated genotypes. (C) Schematic drawing of a middle S9 egg chamber illustrating the BCs (yellow), NCs (gray), FCs (purple), BM (green), the mirror region (pink square) and the positions where AFM measurements were taken, anterior to the mirr region (A, black arrow) and in the mirr region (C, pink arrow). (D, E) Comparison of the apparent elastic modulus K in the mirr region (C) and anterior to the mirr region (E), in 4 control mirrGal4 (D) and mirr>EHBP1mCh egg chambers. A minimum of 4 different readings (circles)/egg chamber were taken. Horizontal lines in B, D, and E represent mean values. The statistical significance of differences was assessed with a t test, * P value < 0.05, ** P value < 0.01, and *** P value < 0.001. Horizontal and vertical lines indicate mean and SD, respectively. The raw data underlying panels B, D, and E are available in S1 Data. (JPG) [file pbio.3002172.s016.jpg]

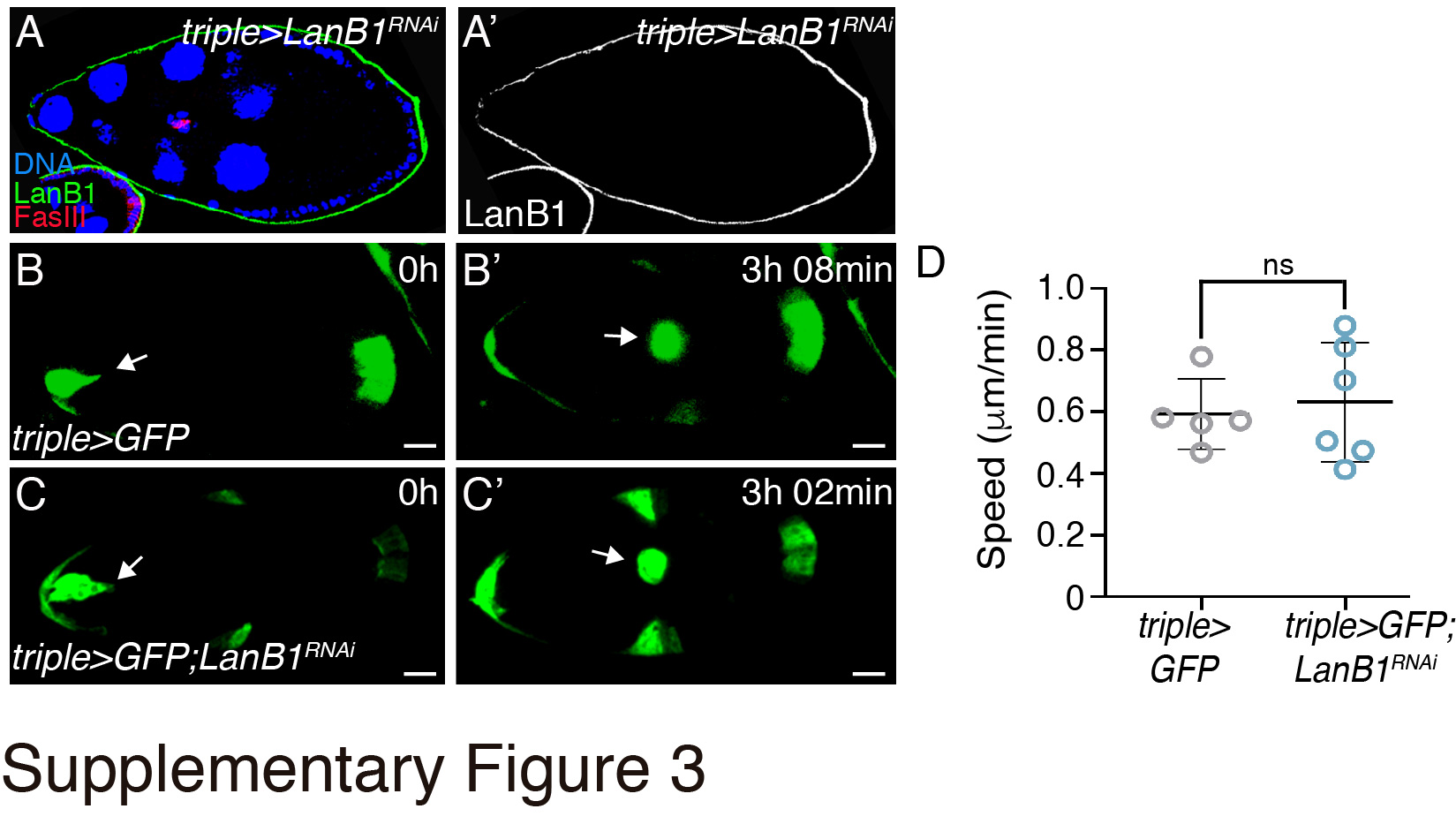

Supplement: S3 Fig — (A, A’) S9 triple>LanB1RNAi (B) egg chambers stained with anti-LanB1 antibody (green), the DNA marker Hoechst (blue) and the PC marker Fasciclin III (FasIII, red). (B–C’) Stills taken from live imaging of BCs from control (C306Gal4; slboGal4; tslGal4) and C306; slbo; tsl>LanB1RNAi egg chambers. (D) Quantification of the migration defects in egg chambers of the indicated genotypes. The statistical significance of differences was assessed with a t test. Horizontal and vertical lines indicate mean and SD, respectively. Scale bars in A” and B”, 20 μm. The raw data underlying panel D are available in S1 Data. (JPG) [file pbio.3002172.s017.jpg]

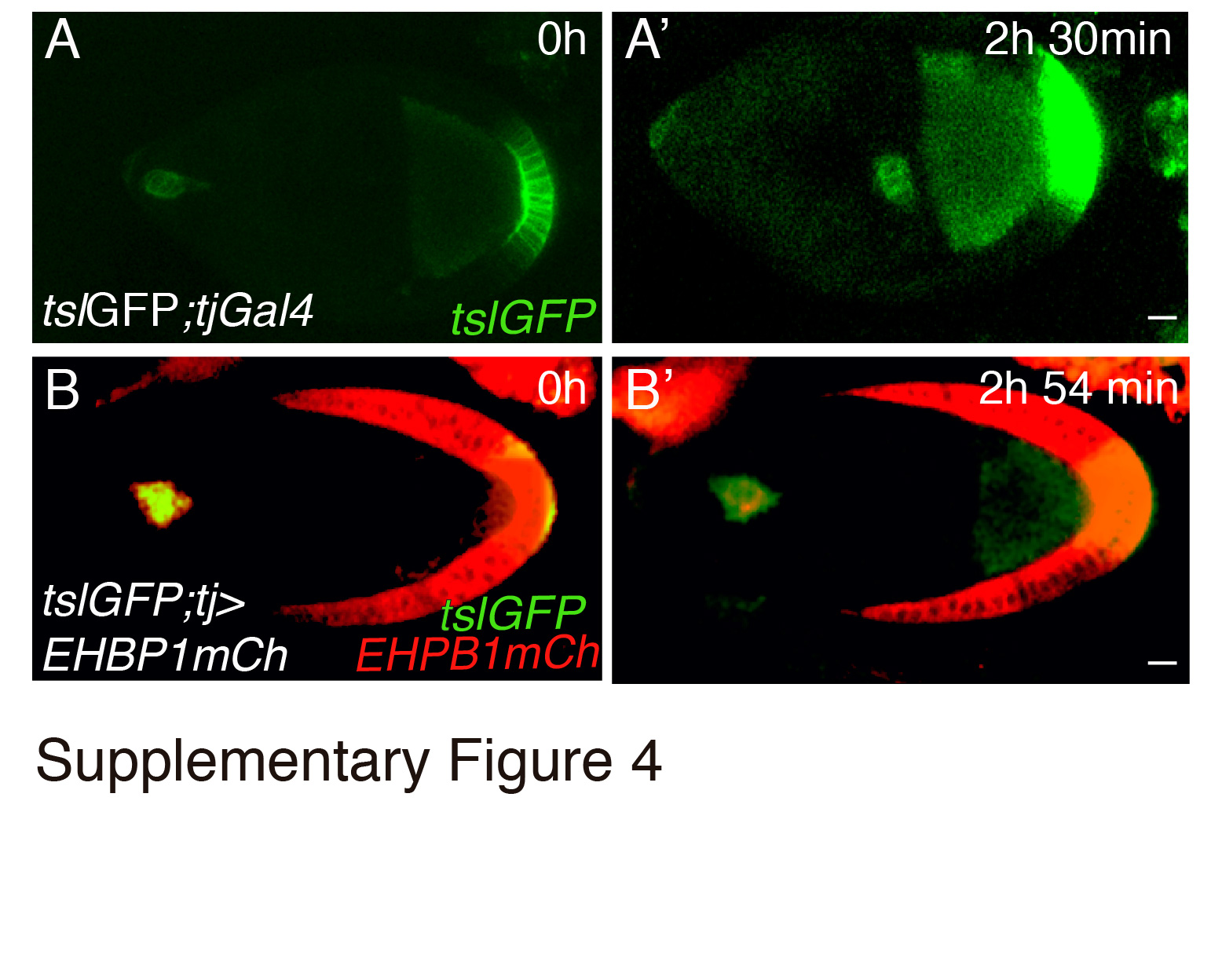

Supplement: S4 Fig — (A–B’) Stills taken from live imaging of migrating BCs from egg chambers of the indicated genotypes. Scale bar in A’ and B’, 20 μm. (JPG) [file pbio.3002172.s018.jpg]

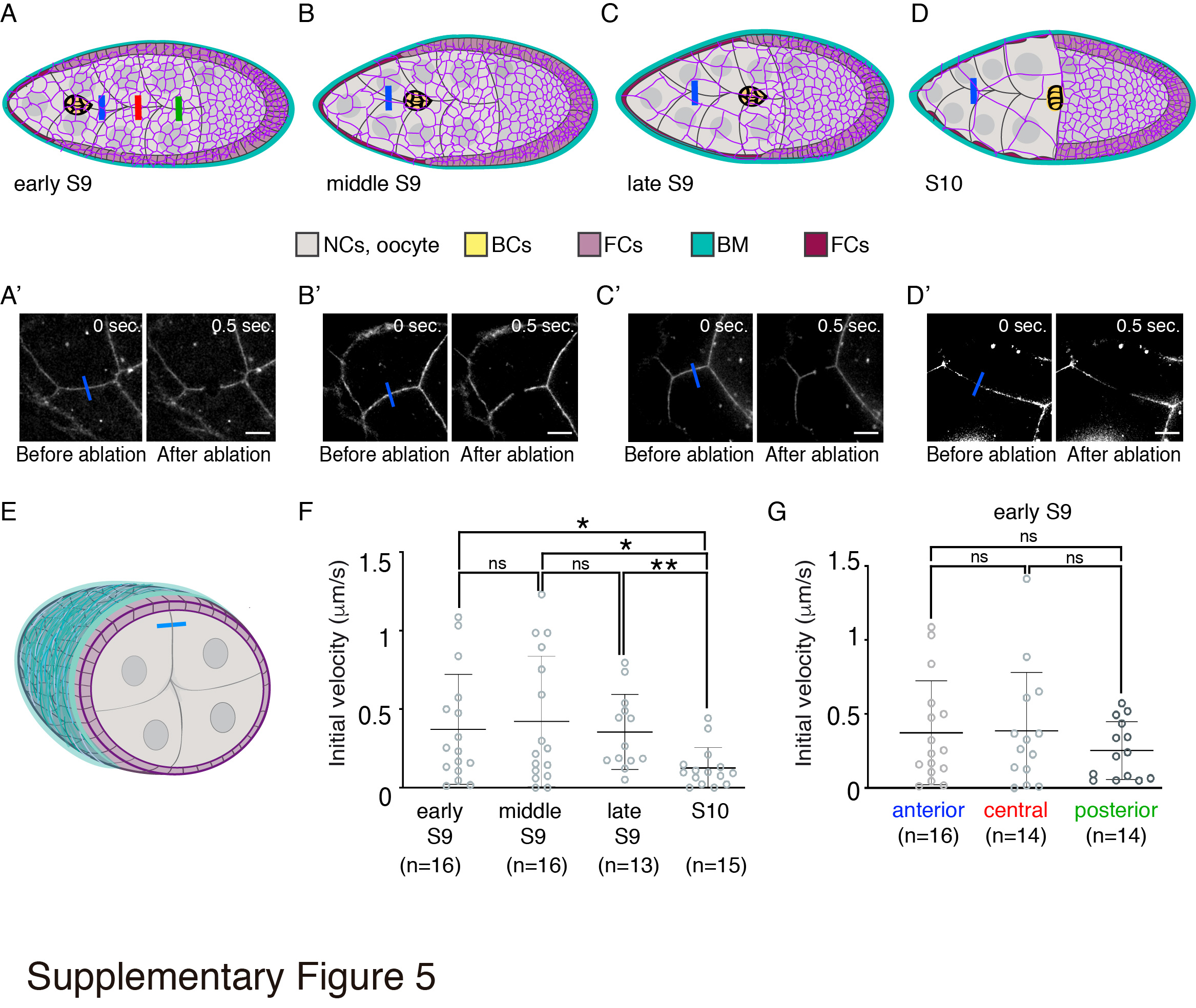

Supplement: S5 Fig — (A–D) Schematic drawings of early S9 (A), middle S9 (B), late S9 (C), and S10 egg chamber illustrating the BCs (yellow), NCs (gray), FCs (purple), BM (green), and the point of ablation in the NCs (blue bar). (A’–D’) Images of life control egg chambers of the indicated developmental stages expressing Resille-GFP before and after NC bonds are ablated. Blue bars indicate points of ablation. (E) Schematic representation of an egg chamber and the point of ablation in NCs. (F, G) Quantification of the initial velocity of vertex displacement of the indicated ablated bonds. The statistical significance of differences was assessed with a t test, * P value < 0.05 and ** P value < 0.01. Horizontal and vertical lines indicate mean and SD, respectively. Scale bar in A’–D’, 10 μm. The raw data underlying panels F and G are available in S1 Data. (JPG) [file pbio.3002172.s019.jpg]

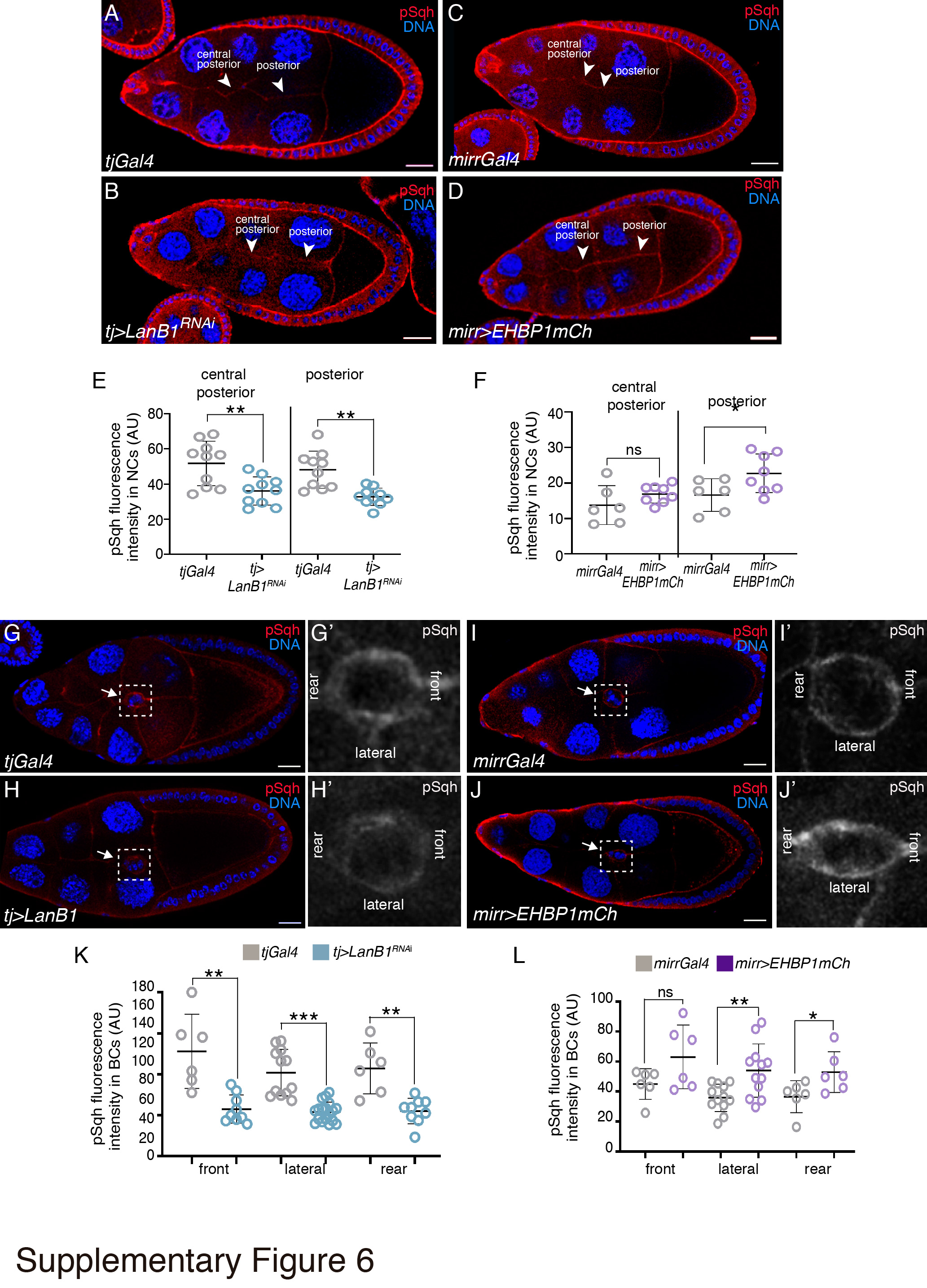

Supplement: S6 Fig — (A–D, G–J’) S9 egg chambers of the designated genotypes stained with anti-pSqh (red) and the DNA marker Hoechst (blue) showing pSqh levels in the NCs (A–D) and the BCs (G–J’). (E, F, K, L) Quantification of pSqh levels in the NCs (E, F) and in the BCs (K, L) of egg chambers of the specified genotypes. G’–J’ Magnifications of the white boxes in G–J. The statistical significance of differences was assessed with a t test, * P value < 0.05, ** P value < 0.01, and *** P value < 0.001. Horizontal and vertical lines indicate mean and SD, respectively. Scale bar in A, 20 μm. The raw data underlying panels E, F, K, and L are available in S1 Data. (JPG) [file pbio.3002172.s020.jpg]

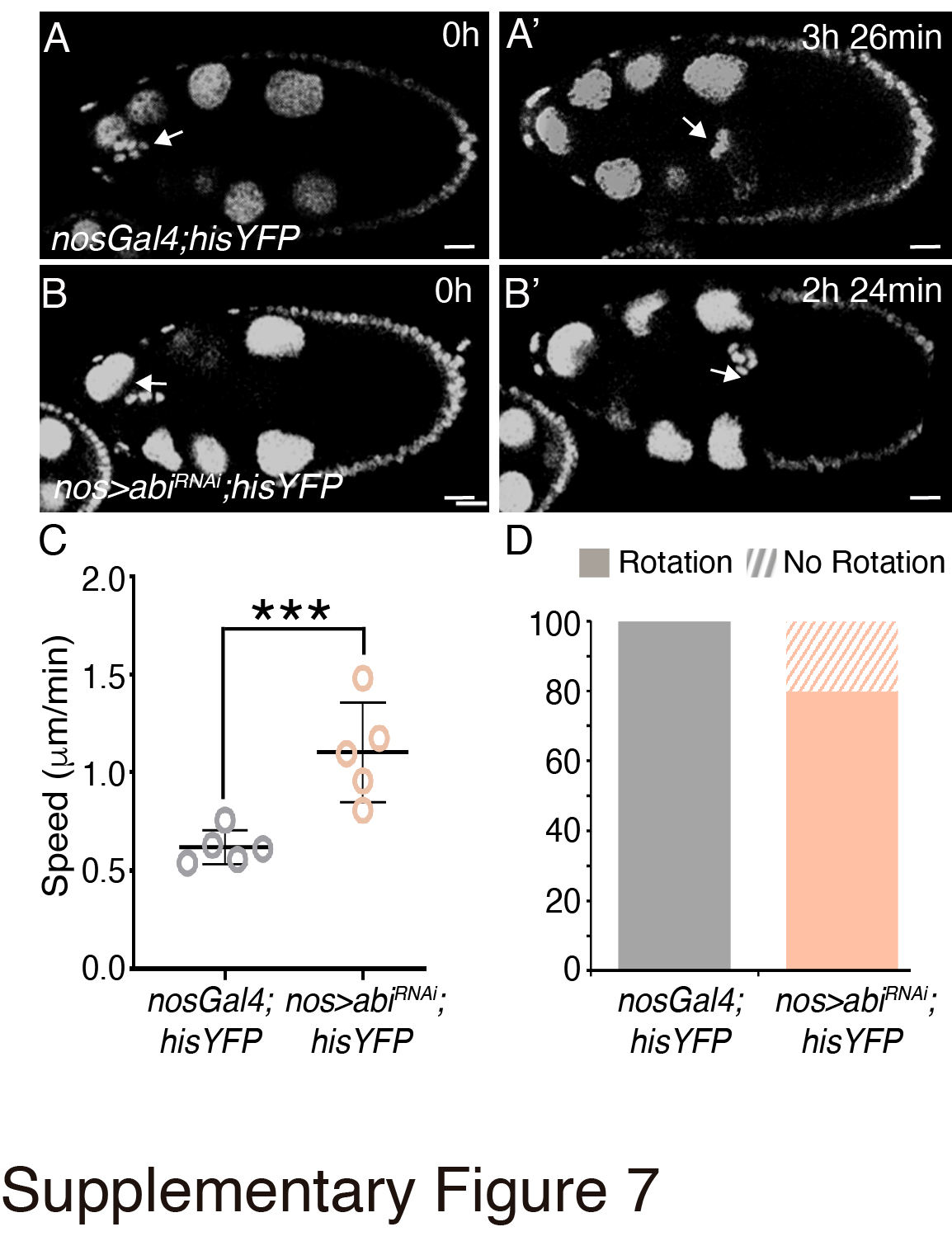

Supplement: S7 Fig — (A–B’) Stills taken from live imaging of migrating BCs from egg chambers of the indicated genotypes. (C) Quantification of the migration defects in egg chambers of the specified genotypes. The statistical significance of differences was assessed with a t test, *** P value < 0.001. Horizontal and vertical lines indicate mean and SD, respectively. Scale bars in A’ and B’, 20 μm. The raw data underlying panel C and D are available in S1 Data. (JPG) [file pbio.3002172.s021.jpg]

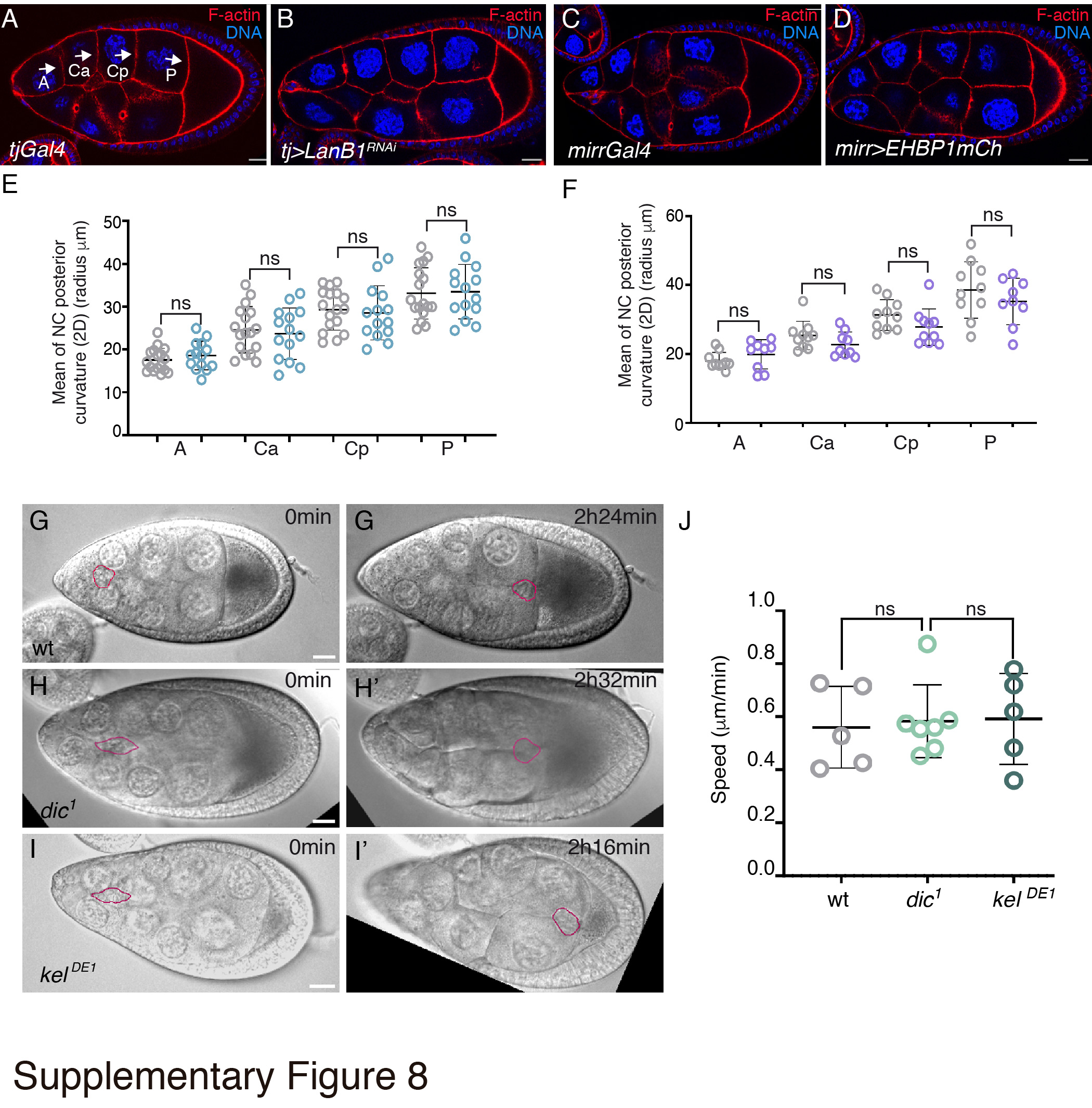

Supplement: S8 Fig — (A–D) S9 controls tjGal4, tj>LanB1RNAi, mirrGal4 and mirr>EHBP1mCh egg chambers stained with the F-actin marker Rhodamine-Phalloidin (F-actin, red) and the DNA marker Hoechst (blue). Arrows in A indicate the curvature of the membranes between an anterior (A) and an anterior central NC (Ca); between 2 central NCs -a Ca and a Cp (a posterior central NC); between a Cp and a posterior (P) NC and between a P NC and the oocyte membrane. (E, F) Quantification of the radius of curvature of A, Ca, Cp and P NCs in S9 egg chambers of the indicated genotypes. BC clusters are marked with a red circle. (G–I) Stills taken from live imaging of migrating BCs from egg chambers of the indicated genotypes. (J) Quantification of BC migration speed in egg chambers of the indicated genotypes. The statistical significance of differences was assessed with a t test. Horizontal and vertical lines indicate mean and SD, respectively. Scale bars in A–D and G–I’, 20 μm. The raw data underlying panels E, F, and J are available in S1 Data. (JPG) [file pbio.3002172.s022.jpg]

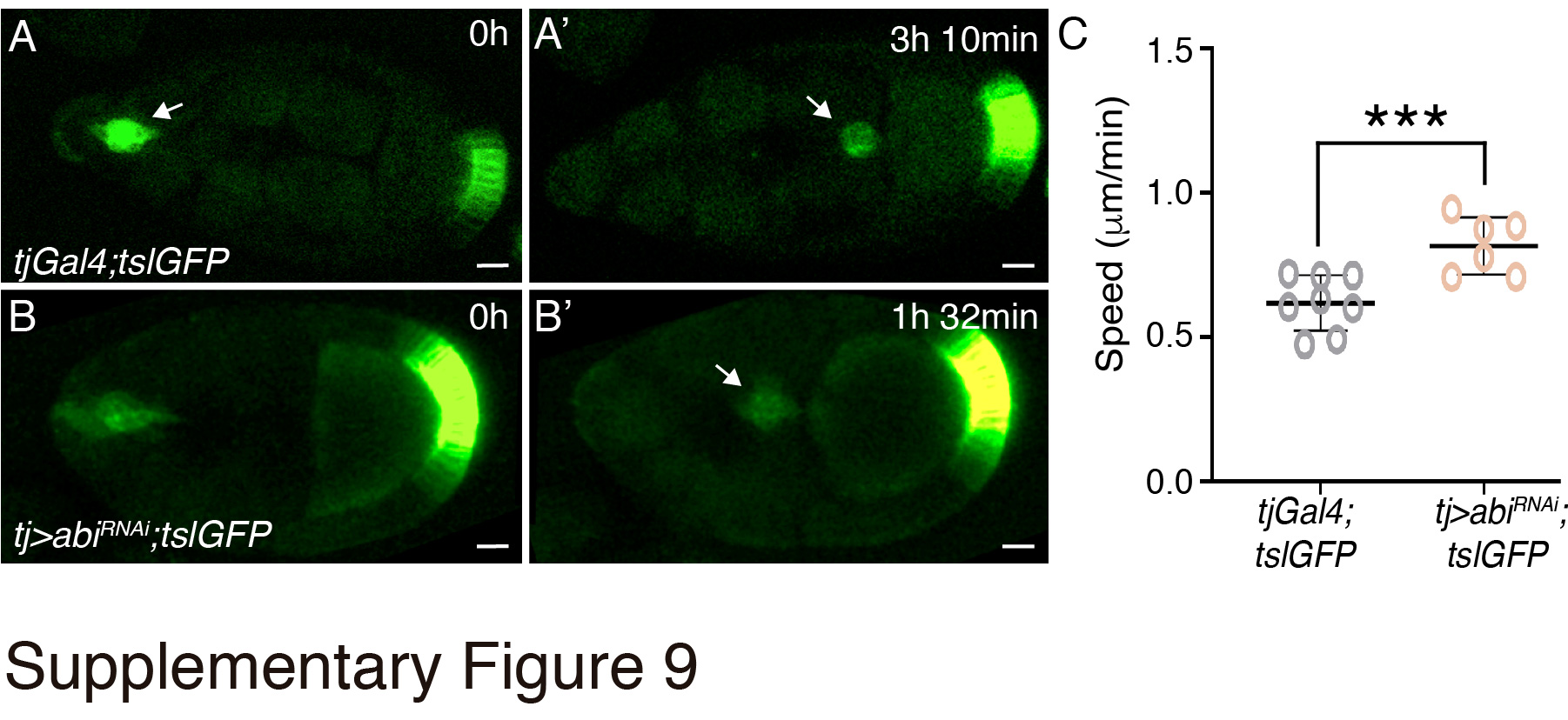

Supplement: S9 Fig — (A–B’) Stills taken from live imaging of migrating BCs from egg chambers of the indicated genotypes. (C) Quantification of the migration defects in egg chambers of the specified genotypes. The statistical significance of differences was assessed with a t test, *** P value < 0.001. Horizontal and vertical lines indicate mean and SD, respectively. Scale bars in A’ and B’, 20 μm. The raw data underlying panel C are available in S1 Data. (JPG) [file pbio.3002172.s023.jpg]
