# Supplementary material for: Constriction imposed by basement membrane regulates developmental cell migration
Source: PLoS Biol. 2023 Jun 28;21(6):e3002172. doi: 10.1371/journal.pbio.3002172 (PMC10335704; doi:10.1371/journal.pbio.3002172)
Supplement: Attachment — Submitted filename: 132663_1_rebuttal_2620562_rr7yxv.docx [file pbio.3002172.s025.docx]

**Manuscript number:** #RC-2022-01792

**Corresponding author(s):** Maria D, Martín-Bermudo

*[Please use this template only if the submitted manuscript should be considered by the affiliate journal as a full revision in response to the points raised by the reviewers.*

*If you wish to submit a preliminary revision with a revision plan, please use our "*[*Revision Plan*](https://docs.google.com/document/u/0/d/1HHp62Tfeb44nd3Gu_3ChB0b8gmMdEog7GjZcZFwHSiQ/edit)*" template.* ***It is important to use the appropriate template to clearly inform the editors of your intentions.****]*

## 1. General Statements [optional]

*This section is optional. Insert here any general statements you wish to make about the goal of the study or about the reviews.*

Mechanobiology, and in particular that of basement membranes, is an exciting and timely area of study, arising from the intersection of biophysics/engineering and genetics. In fact, there is increasing interest in how the basement membrane alters forces within tissues and how these forces impact on the morphogenesis and homeostasis of tissues. In this work, we have combined genetics with live cell imaging, laser ablation and biophysical measurements, such as atomic force microscopy, to relate, for the first time, basement membrane mechanics to migration via constriction and cortical tension. In this revised version of our manuscript, we have addressed all of the reviewers´ concerns and suggestions (see comments to all referees). We believe that the included changes increase the quality, interpretation and significance of our findings. We hope that the reviewers will agree that the new results significantly improve the manuscript in a manner suitable for publication in a journal with a developmental and cell biology audience, such as PLoS Biology. We take this opportunity to thank the Reviewers for their efforts and for very useful comments.

## 2. Point-by-point description of the revisions

*This section is mandatory.* *Please insert a point-by-point reply describing the revisions that were already carried out and included in the transferred manuscript.*

**Response to Reviewers**

**Reviewer #1** (Evidence, reproducibility and clarity (Required)):

*The manuscript by Lopez et al have employed the ex vivo model of Border cell migration in Drosophila ovaries to examine the constriction forces imposed by basement membrane on migrating border cells. The authors have extensively employed live cell imaging coupled with genetics to demonstrate that basement membrane encasing fly eggs modulates the dynamics of migrating border cells. Through laser ablation experiments they show that basement influences the tension of the underlying follicle cell and nurse cells which in turn affects the migration efficiency of collectively moving border cells. Over all the experiments are well quantified with good degree of statistics that drive the claim of the authors.*

*Major comments: Over in all the images, it is very hard to appreciate the overall contour of the egg chamber. This is important to get an insight regarding the stage of the egg chamber being evaluated.*

We agree with the reviewer in that, in some cases, it is difficult to appreciate the overall contour of the egg chamber. This applies mainly to still images taken from movies. This is mainly due to the fact that normally the fluorescent settings are set to properly visualize BCs as they move, leaving other areas of the egg chamber a bit out of focus and/or with a much weaker signal. Nonetheless, we have increased the contrast in the movies, so that the outline of the egg chambers can be better visualized in the figures. In addition, the desired stages were always selected using bright field before recording the movies.

*1. By depleting the constituents of basement membrane, the authors show that the speed of the migrating border cells increases. However in Supplementary Figure 1A and B where that authors have depleted LanB1, the migrating border cell cluster seems to lag while the control has reached the oocyte boundary. Is this a single off phenomena?*

Yes, this is an unfortunate single off phenomena, we are sorry about this. We have now changed this image to a more representative one.

*2. This is regarding the osmotic swelling experiments. The frequency and speed of bursting of egg chambers in deionized water was used to evaluate the stiffness of basement membrane in different genetic background. As egg chambers of different stages have variable sizes, it would be fair to evaluate egg chambers of only a particular stage for this analysis as the tonocity of the egg chambers may depend on their size.*

We believe, in agreement with this reviewer and reviewer 2, that the osmotic swelling assay is not the most accurate method to measure BM stiffness. Thus, we have now performed atomic force microscopy (AFM) measurements and have incorporated this data in a new Fig.S2. The AFM measurements confirm the results obtained from the osmotic swelling assay, showing that reducing or increasing the levels of BM components decreases or augments BM stiffness, respectively.

*3. Line 211, "Live time lapse imaging, showed that the overexpression of EHBP1mCh in all FCs delayed BC migration (tslGFP; tj> EHBP1mCh, Figure S4A-B', Movie S4, n=6)." Though the border cell cluster hasn't moved significantly in Fig S4B', the egg chamber development seems to be stalled as the movement of main body follicle cells is affected. My concern if over expression of EHBP1mCh in the follicle cells is stalling the oogenesis itself could that indirectly affect the border cell movement.*

We agree with the referee in that overexpression of EHPB1mCh in all FCs, using the *tjGal4* line, might be affecting the stretching of the anterior FCs and, hence, indirectly, BC migration. This combined with the fact that the *tjGal4* line drives expression in all FCs, including BCs, prompted us to use the *mirrGal4* line, which drives expression specifically in the central FCs and does not affect anterior FC stretching. Furthermore, we show by AFM, in this new version of the manuscript, that overexpressing *EHBP1mCh* specifically in the central region, using the *mirrGal4* line, increases BM stiffness locally in this region (new Fig.S2). Our analysis of BC migration in this experimental situation shows that, while BCs migrated normally anterior to the *mirror* region in the *tslGFP; mirr>EHBP1mCh* egg chambers, migration speed was reduced once BCs reached the *mirror* region (Fig.2), strongly suggesting that overexpression of EHBP1 slows BC migration, most likely by increasing BM stiffness.

*Secondly though EHBP1 has been shown to affect secretion of the basement membrane constituents, it could also modulate asymmetric secretion of other components. Can the authors evaluate if over expression of EHBP1mCh rescues the delay in migrating border cells in Lanb1 heterozygous background to render stronger support to their claim.*

In the egg chamber, overexpression of *EHBP1* has been shown to specifically increase pericellular Col IV levels and BM fibril fraction and length, without affecting Col IV levels (Isabella and Horne-Badovinac, Dev. Cell 2016). Thus, we also thought, in consonance with the reviewer, that the ectopic expression of *EHBP1* could rescue the BM stiffness defects due to laminin downregulation, and, hence, BC migration. However, we had previously shown that Col IV (and Perlecan) levels in egg chambers depleted of Laminins were reduced by half compared to control (Diaz de la Loza et al, Cell Reports, 2017). In this context, the local ectopic expression of *EHBP1* in laminin-depleted flies would result in follicles with low levels of Laminins and Perlecan and a slight increase in Col IV fibrils in the central region, which we were not sure would be enough to rescue the BC migration defects detected in laminin-depleted follicles. In any case, we did try the rescue experiment, but, unfortunately, it turned out to be impossible to perform. First, we could only express *EHBP1* in the central region of the follicle, using the *mirrGal4* line, as its expression in all FCs, using the *tjGal4* line, would cause defects in stretch and BCs, which would indirectly affect BC migration (see above). Second, in order to express *EHBP1* in the central region of laminin-depleted flies, we could not use the *tj>LanB1^RNAi^* condition, as this would result in the expression of *EHBP1* in all cells, including stretch and BCs, which could lead to defects indirectly affecting BC migration, as mentioned above. Thus, we used as laminin-depleted background another condition used in this study, the combination of the two laminin alleles *LanB1^28a^* and *l(2)K05404*. However, the local expression of *EHBP1*, using the *mirrGal4* line in *LanB1^28a^/l(2)K05404* flies, which are already quite sick, turned out to be lethal. Thus, unfortunately, we have not been not to perform the rescue experiments with the available tools. In any case, we believe that the new AFM results demonstrating that reducing the BM components Laminins and Col IV or increasing *EHBP1* levels, which results in a decrease and an increase in BM stiffness, respectively, correspondingly correlates with reduced and increased BC migration speed, strongly supports our hypothesis that the stiffness of the BM membrane influences BC migration.

*4. In Supplementary Fig 7 B and B' the nurse cell morphology seems to be affected. Could the distorted nurse cell morphology in the abi-depleted germline cell affecting the migration efficiency of border cells.*

The images shown in Fig.S7 are stills taken from live imaging of migrating BCs from egg chambers of the indicated genotypes. As for live imaging egg chambers are in suspension in culture medium, sometimes not all NCs are properly focused, given the impression of aberrant NC morphology. We have now chosen another movie in which more NCs are in focus, which shows that NCs have normal morphology. In addition, we have changed the images in Fig.S7 B and B', with stills taken from the new movie.

*5. Line 313-314 The authors state that "The radius of curvature of a spherical interface is inversely proportional to the difference in pressure between the two sides of the interface." This may be applicable to a smooth surface but may be not directly applicable to the cell membrane as there are local regional variations and thus any inference on the cytoplasmic pressure of nurse cells may be misleading.*

Although we agree with the reviewer in that the radius of curvature might not be the most accurate method to measure cytoplasmic pressure, this was previously proposed to reflect NC mechanics (Lamire et al. PLoS Biology 2020). Thus, it was reasoned that the extent of curvature of the outward-facing NC membrane (the radius of the circle fitting this membrane) could indeed reflect NC mechanics. In this context, it was assumed that the radius of curvature of a spherical interface was proportional to the ratio of its tension and inversely proportional to the difference in pressure between the two sides of the interface: R = 2γ/ΔP, which could be generalized to non-spherical interfaces by replacing 2/*R* by the mean curvature of the interface. Using this method, a gradient of cytoplasmic pressure along the A/P axis, with pressure decreasing from anterior to posterior, was reported. Interestingly, this gradient was still detected upon treatment with collagenase, in agreement with our results showing presence of the gradient in laminin-depleted follicles. This is why we decided to use the radius of curvature as a first approximation to analyse a possible contribution of NC cytoplasmic pressure to BC migration. In addition, as Lamire et al. used this method to show that *dic* mutation led to a decrease in pressure while *kel* mutations yielded an increase in pressure, we also analysed BC migration in these mutants and showed it was not affected. However, we agree with the reviewer in that our results should be treated as preliminary data, and this is why we have stated in the discussion that “our data suggest that cytoplasmic pressure does not seem to compellingly influence BC migration” and the data is presented in a supplementary figure (Fig.S8).

*Minor comment:*

*1. In supplementary figure 6D, the square boxes are obscuring the border cell membrane and it will be better if the authors can modify the figure to render more clarity.*

We have now changed this supplementary figure and have added high magnifications of the BC cluster for clarity.

*2. There are couple of places where sentence structure needs to be corrected*.

We are sorry about this. We have now gone through the whole text and have tried to correct all typos and sentence structures.

***Referees cross-commenting***

*I agree with all the comments of other reviewers. Overall I also feel that results do not strongly support the main conclusions. The authors draw major conclusions based on experiments that are merely suggestive rather than being conclusive. Some of the concerns are listed. Like Reviewer 3 raising the concern that Collagen IV may have other functions in the basement membrane other than providing stiffness. A similar concern I too have raised regarding over-expression of EHBP1. I agree with Reviewer 3 that there are several other factors that can affect the outcome of bursting assay besides the stiffness of the basement membrane itself. So the authors need to be careful in linking the bursting frequency of the egg chamber with the stiffness of the basement membrane itself.*

As mentioned above, we have now performed AFM measurements, which can measure reliably and quantitatively the stiffness of most tissues and organs, including the BMs.

*I agree with other reviewers that the quality of the images need to be better. In addition, the image presented should be representative of the population and should fit with the over claim made by the authors (Point No 3 of Reviewer 2 and Point No 1 of Reviewer1).*

We have now improved the quality of the images. All images shown are representative of the population, as it is reflected in the quantifications.

*I also agree that authors need to explain Reviewer 2's concern (Point No-1) as to why the lateral protrusion in tj>LanB1RNAi doesn't inhibit the movement of border cell clusters but rather produce faster migration speeds?*

Please, refer to our reply to Reviewer 2's concern (Point No-1) below.

*Lastly it is important for the authors to verify that like Kel/Dic mutants are indeed effective or any genetic perturbation like overexpression of EHBP1mCh is not the stalling the oogenesis progression perse, thus giving a false impression of altered migratory speed of border cell clusters.*

Please, consult our reply to Reviewer 2's concern (Point No-2) below.

*Reviewer #1 (Significance (Required)):*

*The role basement membrane is well documented in affecting the shape of neighbouring cells Here the authors claim that the stiffness of basement membrane is regulating the migration efficiency of the border cells. I believe that basement membrane encasing the follicle and underlying germline cells provides a very narrow passage for the border cells to migrate. Any mechanical perturbation that releases or increases the pressure or make the nurse cells membranes less or more taut will affect the dynamics of migrating border cells. Though the authors have demonstrated this with very elegant experiments, I am afraid that their findings are standard outcomes in any physically constrained system and somehow doesn't significantly advance the field.*

We agree with the reviewer in that any mechanical perturbation that make the NC membranes less taut or tauter will affect the dynamics of migrating BCs. However, what it was not known is that the stiffness of the BM, not being in direct contact with the NCs, could affect their mechanical properties and, hence, the migration of BCs located at several cell layers distance from the BM. Thus, in agreement with reviewers 2 and 3, we believe the concept is quite novel and fills the indeterminate relevance of the surrounding environment on collective cell migration.

***Reviewer #2*** *(Evidence, reproducibility and clarity (Required)):*

*Ester and her colleagues described a force model that controls border cell migration by varying the stiffness of the basement membrane. It's based on the modification of laminin and Coll IV which are components of the basement membrane. To reduce BMs stiffness, they introduced LanB1RNAi or the LanB1 mutant to inhibit laminin production or vkgRNAi to reduce Coll IV. They also applied EHBP1mCh to enhance BM stiffness. Furthermore, they applied laser ablation to confirm that the BM stiffness affects the tension of nurse cells and follicle cells, thus regulating border cell behavior by changing environmental properties. It is a nice work revealing how the environment controls border cell migration; however, there are several points that concern me:*

*1. It's reported that actin polymerization at the front of the cell generates protrusions, as well as that myosin contractility helps to suppress lateral random protrusions, thus leading to a directed and efficient cell migration. So why do more lateral protrusions (tj>LanB1RNAi) produce a faster migration speed?*

Based solely of imaging fixed tissue, Fulga and Rørth (2002) suggested that protrusions function as a grapple to pull the cluster forward (Schober and Perrimon, 2002). This model implied that the tip of the protrusion adhered strongly to the substrate and would not retract but rather would be incorporated into the advancing cluster. However, a study, analyzing protrusion dynamics by live imaging, showed that most protrusions retracted, suggesting that most protrusions were not effective grapples (Mishra et al. MBoC, 2019). This study also demonstrated that protruding clusters moved with similar velocity that retracting ones. From this analysis, it was concluded that protrusions are dynamic and rather than functioning as attachment sites, they function as sensory organs. In this context, it was also proposed that protrusions encountering a more favorable environment, such as physical openings, would extend further before retracting. In addition, another study from this group showed that protrusions would extend in the direction of more advantageous environment, such as at the junctures where several NCs meet (Dai at el. Science, 2020). In this context, the orientation and number of protrusions do not necessarily have to correlate with migration speed and this could explain why we observed faster migration despite the increase in lateral protrusions. In fact, it is in this scenario where we believe we can explain our results. Thus, in the discussion we propose that, in contrast to what happens in controls where projections are normally form at the front, where the environment is more favorable, the reduced NC cortical tension we observed in laminin-depleted follicles could lead to a lessening of cellular junctions, which would increase the frequency of lateral projections. We have now extended our discussion to explain our hypothesis better.

*2. We know some labs also did experiments with those Kel/Dic mutant flies. And the Kel mutant is very sick, which sometimes leads to NC degeneration. As a result, we have serious doubts that this mutant's border cell migration will remain normal.*

Indeed, we also found some *Kel* mutant egg chambers in which NCs degenerated. However, we discarded these egg chambers and only analyzed those in which the defects associated to the *kel* mutation, such as small oocyte, were still present, but egg chambers looked healthy and developed normally, as judge by NC morphology and behavior and retraction of the FE (see Movie S10).

*3. From figure4, we noticed that with mirrGal4, the vertex distance increase is much lower than tjGal4 (control of D, H and K), and even with expressing the EHPB1mCh, the distance is still lower than the tjGal4 control. These indicate the NC cortical tension is lower with mirrGal4 expression, which is partially against the paper's main point. (Similar issue in figure5 D and E).*

Indeed, the vertex distance increase is much lower in the *mirrorGal4* than in *tjGal4*. We attribute this result to the fact that these two controls have different genetic backgrounds. This is why it is so important to always measure the appropriate control for each experimental condition. We do not believe one can compare the measurements from *mirr>EHBP1mCh* with those from *tjGal4* follicles, rather *mirr>EHBP1mCh* should be compared with its own control, *mirrGal4*, as the genetic background could influence the mechanical properties of the tissue, as it seems to be the case. In fact, when we were measuring the NC cortical tension of *tjGal4* and *tj>LanB1^RNAi^* follicles, we also measured that of *tj>EHBP1mCh* and the results showed that NC cortical tension was increased in *tj>EHBP1mCh* compared to both *tjGal4* and *tj>LanB1^RNAi^*:

We did not include this result in the manuscript because at that point, we had already decided to use the *mirrGal4* line to overexpress EHBP1mCh in the FE, to avoid indirect effects of its expression in BCs when using the *tjGal4* line. However, we will be happy to include this result in the paper should the reviewer consider it necessary.

*4. Sfigure1 A and B seem not to have the right contrast (the blue and the red should have the same brightness), so the comparison of the intensities might be inaccurate and needs to be requantified after adjustment of the images.*

The reviewer is right and we apologize for this. In addition, the egg chambers shown in Fig.S1A and B are different stages. Thus, we have now repeated the staining and have made new quantifications in S10 egg chambers, using the same confocal settings (New Fig.S1).

*5. Sfigure2 A-E showed that the vkgRNAi has the highest bursting frequency, whereas F and G do not. And the majority of the data from F does not fit with A-E, and it is unclear what timepoint sF.G. is at.*

In collaboration with the lab of Kristian Franze (University of Cambridge, UK) and I. Palacios (QMU, London), we have performed atomic force microscopy measurements, as we believe this to be a more accurate method to measure BM stiffness. This new data has been incorporated in a New Fig. S2. We believe our new AFM measurements, which are in agreement with those obtained from the osmotic swelling assays, convincingly prove that reducing or increasing the levels of BM components decreases or augments BM stiffness, respectively.

*6. SFigure 6 only displayed a representative image of the control condition; the lack of representative images for the other conditions resulted in unconvincing results.*

We have now generated a new Fig.S6 containing representative images for all conditions.

*7. Some figures and movies have prominent variation of migrating stages, such as not-detached border cells compared with detached border cells. This might strongly cause the results inconsistent with each other.*

The capture of the whole process of BC migration, just before they detach from the FE until they reach the anterior membrane of the oocyte, always is quite difficult. However, we do not need to do this to calculate the speed of BC migration. What is needed is to always measure the speed for a similar length of time and in the same region of interest in controls and experimental conditions, either most of the migratory process, when we manipulate BM stiffness in the whole egg chamber (using the *tjGal4* line), or from the middle region to the end, when we manipulate the stiffness of the BM only in the central region (using the *mirror Gal4* line). And this is how the movies and quantifications were performed.

*8. There are numerous typos in both the manuscript and the figures.*

We are sorry about this. We have now gone through the whole text and have tried to correct all typos in both the manuscript and the figures.

*Based on all these concerns, I recommend authors to do some improvement before this manuscript is accepted by some reputed journals.*

In the new modified version of the manuscript, we have addressed all of the reviewers´ concerns and suggestions (see comments to all referees). We believe these improvements enrich the manuscript in a manner which is now suitable for publication in a reputed journal with a developmental and cell biology audience, such as PLoS Biology.

*Reviewer #2 (Significance (Required)):*

*Strengths: the manuscript is well written and organised; limitation: figures and results are not supportive enough, and thus conclusion is not completely convincing, statistical quantification is not clear and somehow confusing.*

*Advice: If the conclusion is solid, this story will fill the unclear importance of surrounding environment on cell-rich tissue for collective cell migration. Concept is very novel while needing more supporting data. It is a fundamental study for development biology.*

*Audience: The story will fit well for developmental and cell biology, as well as people with biomechanical background.*

In this revised version of the manuscript, we believe we have addressed all of the reviewers´ concerns and suggestions (see comments to all referees). One of the main concerns of the reviewers is whether the defects in BC migration we have found in our experimental conditions where we manipulate the levels of BM components are indeed due to changes in BM stiffness, as the method used to measure BM stiffness, an osmotic swelling assay, is not an accurate one. In this new version of the manuscript, we have used AFM which is a reliable and quantitative method to measure stiffness of tissues, including BMs. These measurements show that indeed our approach to change BM component levels leads to changes in BM stiffness. We believe these results strongly support our conclusions that BM mechanics regulate migration via constriction and cortical tension. We have also improved the text by editing when necessary, we have extended the discussion to accommodate the reviewers´ concerns, we have added a new Figure (Fig.S2) and we have modified all main Figures, except Fig.7, and all Supplementary Figures, to show images and quantitative data in a clearer way (all bar charts have been changed to graphics showing individual data points with means and s.d., as suggested by Reviewer 3, see below). As we always compare to samples, control versus experimental condition, the statistical analysis was done using Student´s *t*-test. In addition, outliers from data sets were identified by the ROUT method with Q= 1% and were not included in the analysis and graphs.

We would like to take this opportunity to truly thank the considerable effort reviewers have put in revising our work and for very useful comments. We believe that the included changes have definitively improved the quality, interpretation and significance of our findings. We hope that you and the reviewers will agree that the new results significantly improve the manuscript in a manner suitable for publication in a journal with a developmental and cell biology audience, such as PLoS Biology.

***Reviewer #3*** *(Evidence, reproducibility and clarity (Required)):Summary: The manuscript analyzes how the constriction of a tissue by an enveloping basement membrane alters the migration of cells migrating through that tissue. The tissue analyzed is the Drosophila egg chamber, an important model for basement membrane studies in vivo, and the cells migrating through it are the border cells. The border cells migrate through the center of the egg chamber, moving as a cluster between the nurse cells, which are in turn surrounded by follicle cells, which secrete the basement membrane on the outside of the egg chamber. The authors decrease and increase the basement membrane stiffness with various genetic perturbations, and they find that the border cells move more rapidly when the stiffness is reduced. They then investigate how basement membrane stiffness is communicated to the border cells several cell layers inside, by measuring cortical tension with laser-recoil. They found that external basement membrane stiffness alters the cortical tension of the nurse cells and the follicle cells, such that reduced matrix stiffness causes reduced cortical tension; further, reducing cortical tension directly within the cells also results in increased border cell migration rates. They conclude that basement membrane stiffness can alter cell migration in a new way, by altering constriction and cortical tension, with an inverse relationship between stiffness and migration rate. This is a strong manuscript and I would request very few changes.*

*The authors are commended on the rigor and completeness of their study. Several independent methods are used to alter basement membrane stiffness (loss of laminin, knock-down of laminin, knock-down of collagen IV, over-production of collagen IV - all of which end up changing collagen IV levels) and all show the same result. Further, they are extremely rigorous about testing and excluding an attractive alternative hypothesis, that the basement membrane of the border cell cluster itself controls its migration rate. The use of mirror-Gal4 is very elegant and convincing, as it expressed only in the central part of the egg chamber, and they found border cells responded differently only in that region. Moreover, the authors were exceptionally thorough in reproducing the basement membrane mechanical data in their own hands using the bursting assay. Overall, the experimental data support the claims of the paper. There is only one more control I would like to see, for the knockdown of laminin in the border cell cluster with a triple-Gal4 combination. Presumably using all three Gal4 lines was necessary to get complete knockdown, and it would be nice to see anti-laminin for the border cell cluster under these knockdown conditions.*

We have now performed this experiment showing that indeed there is no laminin expression in the BCs in the *triple>GFP;LanB1^RNAi^* follicle, which was somehow expected, as Laminins were shown not to be expressed in BCs during their migration (Medioni and Noselli, 2005) (Fig.S1A and S3A). We decided to perform the experiment just in case there was some laminin expression that was not detectable when using the anti-Laminin antibody.

*Despite the rigor, because all of the manipulations to the basement membrane alter the levels of collagen IV, the authors cannot formally exclude the possibility that collagen IV in the basement membrane has another function besides stiffness, perhaps sequestering a signaling ligand, and that this other function somehow alters the cortical tension of the egg chamber. In the paper by Crest et al, externally applied collagenase served as a control for this possibility, but collagenase will not work for the authors because this study is in vivo. I suggest the authors bring up this caveat in the discussion. If they wanted to extend the study (optional), they could knock down the crosslinking enzyme peroxidasin in the egg chamber, which ought to reduce basement membrane stiffness without changing the collagen content. The problem here is that it hasn't already been shown to work that way in the egg chamber, and so both stiffness and collagen levels would need to be measured. Testing the stiffness directly would be difficult, since the bursting assay is not actually a measurement of stiffness (more on that below). Rather than go this route, I suggest just acknowledging the formal possibility, which seems to me unlikely anyway.*

We have now performed atomic force microscopy (AFM) measurements, which are a reliable and quantitative way to measure BM stiffness, in collaboration with the group of Kristian Franze (University of Cambridge, UK). These measurements show that our approach to decrease or increase the levels of the ECM components Laminins and Col IV indeed results in reduced or enhanced BM stiffness, respectively, strongly supporting our view that the stiffness of the BM contributes to the regulation of BC migration. In addition, we have acknowledged in the discussion the possibility that the stiffness of the follicle BM could influence BC migration in other ways, like regulating the diffusion of chemo-attractants coming from the oocyte.

*In terms of clarity, the manuscript absolutely needs a schematic at the beginning to introduce the egg chamber and border cell migration, labeling the cell types, showing the route and direction of border cell migration, and labeling the A/P axis. Without this the non-expert reader cannot readily understand the study.*

We thank the reviewer for this suggestion. We have added a schematic of the ovaries, the egg chamber and BC migration in an amended Fig.1.

*Finally, in terms of clarity, the authors repeatedly use statements such as "stiffness influences migration rate". Influences how? These results are not intuitive to me, and it would help enormously if the authors would make statements like, decreasing stiffness increases migration (as I tried to in my summary). Here are two examples of statements to refine:*

*• Line 189 - "We found that reducing laminin levels affected the migration speed of both phases (Fig.1F, G)." Please say increased, not affected.*

*• Line 245 -"Altogether, these results demonstrate that the stiffness of the follicle BM influences dynamics and mode of BC migration." Again, be specific about how.*

*There are many such statements, from the abstract to the results to the discussion, where it would help the clarity to be more precise about what kind of influence.*

We have now refined all statements.

*Minor comments: • The movies are beautiful!*

We thank the reviewer for the kind words.

*• All the quantitative data are shown in bar charts with means and errors. It is much better to show the individual data points, superimposing the means and distributions on top of the individual points.*

We have changed all graphics to individual data points and superimposed the means and s.d.

*• The bursting assay does not actually measure basement membrane stiffness; rather, it measures failure after elastic expansion. These are related, as was found by Crest et al and the authors say that at one point, but stiffness and failure are not the same thing. Please change the language discussing this assay to "mechanical properties" rather than stiffness.*

As mentioned above, we have now measured BM stiffness by AFM.

*• The laser-recoil assays are done well and are convincing. Throughout the results section, the authors describe these as measuring "cortical tension", which is correct. However, in the figure legends the language changes to "membrane tension" which is only one component of cortical tension. Change them all to cortical tension.*

We have changed all membrane tension to cortical tension.

*• In the Discussion, it would be nice to include something on the two different modes of migration (tumbling and not tumbling).*

Reducing the levels of non-muscle Myosin II activity in BC clusters has been shown to strongly reduce cluster rotation (Combedazou et al., 2017). This has led to propose that regulation of non-muscle Myosin II is essential for the linear to rotational switch (Combedazou et al., 2017). In this context, the decrease in the rotational behaviour we observed in BCs from laminin-depleted follicles could be explained by the fact that BCs in the mutant egg chambers show reduced non-muscle Myosin II activity. We have incorporated this argument in the discussion.

*• I suggest changing the title to remove the word "forces", because forces are never directly measured from basement membrane.*

This is a fine suggestion and we have changed the title to “Constraints imposed by basement membranes regulate developmental cell migration”.

*• Although Dai et al (Science 2020) is discussed near the end, I suggest bringing this reference up to the introduction, so the reader can have the background on the mechanical aspects of border cell migration at the start of this study.*

We thank again the referee for this adequate suggestion. We have now discussed this mechanical aspect of BC migration in the introduction.

*• Two typos (there may be more): At the bottom of Fig. 2, text turns strangely white that should probably be black; and in line 260, you mean Fig. S5 not S4 (laser ablation).*

These typos have been corrected.

*Reviewer #3 (Significance (Required)):*

*Mechanobiology, and mechanobiology of the basement membrane, is a vibrant area of study now, arising from the intersection of biophysics/engineering and genetics. There is general interest in how the basement membrane alters forces within the tissue, and this study is the first to my knowledge to relate basement membrane mechanics to migration via constriction and cortical tension. The authors do a great job of discussing the broader significance of their work in the Discussion. To greatly broaden the scope of this work in the future, the authors could collaborate with a mouse team to look for similar responses in a mammalian tissue, as they discuss.*

*It is worth noting that there is a lot of work on matrix stiffness and migration showing that stiffness promotes migration speed; in these cases, matrix is a substrate, not a compression mechanism. But the opposite nature of the result in interesting and makes this work non-intuitive and perhaps hard for some readers to grasp.*

*As the paper is written now, I think the audience for this work would mostly be oogenesis, border cell migration, and/or basement membrane researchers in the Drosophila community, of which there are many (I am in this camp). With some rewriting to make it more accessible to other audiences, I think it would be interesting to a larger developmental biology audience. The content is not like any other paper I know, but it may be similar in scope and subject matter to the papers detailing how follicle cells and basement membrane interact during follicle rotation.*

We appreciate the reviewer’s words regarding our work and thank for the appraisal of our manuscript and for interesting comments and suggestions. We believe that the included changes increase the quality, interpretation and significance of our findings. We hope that the reviewers will agree that the new results significantly improve the manuscript in a manner suitable for publication in a journal with a developmental and cell biology audience, such as PLoS Biology.
