# Supplementary material for: Constriction imposed by basement membrane regulates developmental cell migration
Source: PLoS Biol. 2023 Jun 28;21(6):e3002172. doi: 10.1371/journal.pbio.3002172 (PMC10335704; doi:10.1371/journal.pbio.3002172)
Supplement: Attachment — Submitted filename: Response to Reviewers.docx [file pbio.3002172.s027.docx]

**Response to Reviewers**

***Reviewer #1:*** *The revised manuscript by Lopez et al is much better specially with the inclusion of Atomic force microscopy data. The authors have addressed most of my concerns satisfactorily. However, I do have some concern regarding the choice of word in the manuscript and seek some clarifications listed below.*

*1. I have a concern regarding the word "regulate" in the title, abstract and text. Since the basement membrane is affecting the kinetics of border cell migration by modulating the cortical tension of nurse cell and follicle cells, I would believe this effect is indirect. Thus it would be appropriate to use the word "affect" or "modulate" in the title, abstract and text over the word "regulate”. The title may read as "Constraints imposed by basement membranes modulates developmental cell migration”.*

Following advice from the editor and Reviewer 2, we have changed the title to: “Constriction Imposed by Basement Membrane Regulates Developmental Cell Migration”.

*Some minor comments:*

*1. I do seek some clarification in Supplementary Figure 2D and 2E. What do C1, A1; C2, A2; C3, A3 and C4, A4 denote? Are these a set of readings for different points in region C and A? Secondly, for each point like C1 are the circles denoting one reading? Figure legend can include more information to render clarity on this.*

The reviewer is right in that the data in this Sup. Fig. needs more clarification. Cs and As are pairwise comparisons of different measurements (circles) in regions C and A of four different control (D) and experimental (E) egg chambers. We have added this information to both the figure and the figure legend.

*Secondly in the materials & methods section under "Atomic Force Microscopy measurements" the authors are using the word ovariole (Line 765-768) instead of egg chamber.*

We have now changed the word ovariole for egg chamber.

*2. Line 225 "Thus, even though Laminins are not expressed in.." Delete word "Thus".*

Done.

***Reviewer #2*** *(Andrea Page-McCaw, signs review): I think the significance of this work warrants its publication in PLoS Biology, as it reveals a new, important, and generalizable mechanism by which the stiffness of basement membranes influences cell migration.*

*I had very few requests on the previous round of review at Review Commons, and I am generally satisfied that the paper is ready for publication. I have a few points for the authors and editor to consider together:*

*1. I recognize that the authors changed the title at my request, but I think "constraint" is too vague. How about "Constriction Imposed by Basement Membrane Regulates Developmental Cell Migration"*

We have followed the advice of this reviewer and that from the editor and changed the title to “Constriction Imposed by Basement Membrane Regulates Developmental Cell Migration”.

*2. Give the sample number in Fig. 3K,L on the figure or in the legend.*

Sample number in Fig. 3K, L has been included in the figure.

*3. There are still some cases when I wish the authors were specific about the effect, rather than saying "affected" and "influenced". Examples are lines 213, 217, 293, 295 (line numbers from the marked up manuscript).*

We have looked for those cases where we can be more specific about the effects and have changed the text accordingly, i.e. examples pointed out by the reviewer and lines 215, 288, 382 and 394 and headings of Figure Legends 4 and 5.

*4. Although the developmental changes in cortical tension are interesting, they do not demonstrate that BM affects that cortical tension - there could be many changes happening in development. The direct manipulations of the BM are sufficient to address this question. I suggest softening the word demonstrated in line 317.*

We have changed the word demonstrated for suggested.

*5. The experiments on cytoplasmic pressure should be presented without so much attention on the A-P gradient of pressure and more on the absolute pressure. The A-P gradient of pressure is not oriented correctly to explain either BC migration or to transmit the constriction of the basement membrane; however the absolute pressure does both. I don't find the radius of curvature to be relevant because it only reports the difference in pressure. The relevant experiments here are dic and kel mutants, which Lamire et al 2020 showed to have reduced pressure by about 4-fold (dic) and increased pressure by about 30% (kel). The authors do not find a change in BC speed in these mutants, and that is relevant. But I still think the authors should be just a bit cautious about their interpretation, considering that Lamire et al 2020, defined cytoplasmic pressure as "the force that counters cortical tension". Since Martín-Bermudo and colleagues show that cortical tension is modulated by the basement membrane and is important for BC migration, it appears on the face of it that cytoplasmic pressure would also be important, and Lamire did find that collagenase reduced cytoplasmic pressure of nurse cells by about 6-fold.*

We agree with this suggestion, which, in a way, we had already contemplated, as we had ended the section of the results dedicated to this aspect with the following sentence: “All together, these results suggest that NC cytoplasmic pressure **on its own** does not seem to strongly contribute to the forces regulating BC migration.” However, in light of this suggestion we have further softened our argument in the results section by changing the subheading from “**The BC migration defects associated to changes in BM stiffness are not due to changes in** **NC cytoplasmic pressure”** to “**Contribution of cytoplasmic pressure to BC migration.”** In the discussion, we convey that cytoplasmic pressure might also be important, but on its own does not seem to be as decisive as BM stiffness. More specifically, the discussion reads as follows: “However, here we show that cytoplasmic pressure on its own does not seem to compellingly influence BC migration. These results suggest that even though the different mechanical properties of cells are interconnected, each of these properties on their own might impact the diverse cell biological processes in distinct ways.”

***Reviewer #3****: Ester and her colleagues tried to address our comments from their previous manuscript. However, their discussion seems not be to supportive while ignoring some previous findings published by other teams. We feel that main conclusion of this manuscript might be true, but the precise mechanism linking the phenotypes (from matrix stiffness to cell tension to border cell migration) might be not precise or fully understood. Thus, we recommend that new submission can be considered again if authors can really address our worried points, as shown below:*

*Major comments:*

*1. The main reference paper (Mishra et al., 2019) in the revised discussion demonstrated that myosin works with E-cadherin to communicate the direction from the lead cell to the followers and that the non-autonomous function and the autonomous function within protrusions need to cooperate to stop follower cells from protruding. This means the lateral protrusions need to be restricted for efficient BC migration, whether they grapple or not. So, main conclusion of Mishra's paper contradicted the hypothesis of currently revised manuscript that lower NC tension leads to a lessening of cellular junctions, which increases the frequency of lateral protrusions, thus leading to a faster BC migration speed.*

Although we agree with the reviewer in that Mishra et al. propose in their work that myosin cooperates with E-cadherin to communicate the direction from the lead cell to the followers and that the non-autonomous and autonomous functions within protrusions need to cooperate to restrict follower cells from protruding, thus allowing directional persistence, they also suggest that most protrusions were not effective grapples. And it is in this context, and taking into account that another work from the same group showed that protrusions would extend in the direction of more advantageous environment, where we propose that lower NC tension may lead to a lessening of cellular junctions and an increase in the frequency of lateral protrusions. However, we do not propose, as stated by the reviewer, that the “*increase in the frequency of lateral protrusions is what leads to a faster BC migration speed”.* In fact, what we propose in the discussion, especially in the context of protrusions not acting as grapples (Mishra et al., 2019), is that the orientation and number of protrusions do not necessarily have to correlate with migration speed, which could explain our results showing faster BC migration when reducing laminin levels, despite the increase in the number of lateral protrusions. Thus, we disagree with the reviewer in that “*main conclusion of Mishra's paper contradicted the hypothesis of currently revised manuscript”*. Finally, we have found that the length of the ectopic lateral protrusions of BCs from laminin-depleted egg chambers is similar to that of the lateral protrusions found in control BC clusters. This is in contrast to the ectopic protrusions found by Mishra´s et al., when inhibiting myosin activity or expression, which are long and resemble those from the lead cell in wild type clusters, which have been proposed to initiate and guide BC migration. This could explain why BCs from laminin-depleted egg chambers do not loose directionality besides having increase ectopic lateral protrusions. In summary, we do not believe the increase in the number of the lateral protrusions we have found when manipulating BM stiffness have any effect on BC migration speed or directionality, as they are not long and do not resemble the ones at the front of wild type clusters guiding cell migration. We have added the measurements of the length of the ectopic lateral protrusions in control and laminin-depleted egg chambers to the results section and to Figure 3 (new Fig3K) and changed the discussion to better explain our hypothesis.

*2. We also don't agree with authors about the biological role of BC protrusions as a sensory organ, since this discussed point will fight against the traditional function of E-cadherin adhesion between BCs and surrounding NCs. Several papers in the field strongly demonstrated that BC-NC E-cadherin adhesion is permissive for BC migration through between NCs, thus supporting that "grappling" structure should be present in BCs to allow their transient anchorage on the surface of surrounding NCs. Thus, where is "grappling" structure in border cells, if this is not protrusion of BCs? Compared with main body regions of BCs, protrusions have more trend to behave as a "grappling" structure for BC anchorage on NCs.*

The role of BC protrusions as a sensory organ was proposed by the group of D. Montell based mainly on several key observations: 1) 80% of protrusions retract; 2) protruding and retracting clusters move with similar speed and 3) BCs accumulate myosin at the base of fully extended protrusion and then retract the great majority of protrusions before crawling into the space vacated by them. Furthermore, BCs continue to crawl forward even though the protrusion retracts (Mishra et al., 2019). These results led the authors to propose an alternative to the grapple and pull model as the major mechanism of forward advancement and to suggest that rather than function as effective grapples or attachment sites, the tip of the protrusion functions as sensory organs. Then, they propose that “cryptic” lamellipodia observed between BCs (Cliffe et al., 2017) could serve a crawling function. We agree this is an alternative challenging model, which might need to be further demonstrated/refined, and that we should then explain our results also in the context of the grapple model. Thus, we have now changed the discussion and have considered our results in the context of both models. More specifically, the new paragraph in the discussion reads as follows:

“Several studies in the field have led to propose a grapple and pull model for BC migration, by forward-directed protrusions. In this scenario, BCs extend a long protrusion at the front, grip the substrate at the tip and the cell body pulls itself toward the tip, absorbing the protrusion into the cell body (Fulga and Rorth, 2002). Furthermore, the ectopic formation of long protrusions in BCs in different experimental conditions slow BC migration (Aranjuez et al., 2016) (Lamb et al., 2020). Here, we show that BCs from Laminin-depleted egg chambers show increased number of side protrusions, yet they migrate faster. However, we show that these ectopic side protrusions are not long. In fact, they have the same length as side protrusions from controls. This could explain why BCs from Laminin-depleted egg chambers do not loose directionality besides having increase ectopic lateral protrusions. Finally, even though the grapple and pull model is a well-accepted general model for BC motility, an alternative model was proposed, in which rather than functioning as effective grapples or attachment sites, the tip of the protrusion functions as sensory organs. This was based on three key observations: 1) 80% of protrusions retract; 2) protruding and retracting clusters move with similar speed and 3) BCs retract the great majority of protrusions before crawling into the space vacated by them (Mishra et al., 2019). Furthermore, BCs continue to crawl forward even though the protrusion retracts (Mishra et al., 2019). Then, they propose that “cryptic” lamellipodia observed between BCs (Cliffe et al., 2017) might serve a crawling function. In this context, orientation and number of protrusions do not necessarily have to correlate with migration speed, sustaining our results showing that BCs can migrate faster in Laminin-depleted egg chambers, despite the increase in lateral protrusions.”

*3. Authors demonstrated that less NC tension leads to more large protrusions in BCs, which is apparently opposite to some findings of two previous paper (Aranjuez et al., 2016; Lamb et al., 2021): more tension by either RhoGEF expression or Singed loss in NCs can strongly result in an increase of p-MRLC and tension in NCS, thereby leading to enhanced protrusion formation in multiple BCs and slowing BC migration. So, how do authors comment on these conclusions from both published papers to explain the major conclusion (less NC tension results in multiple protrusion formation and faster speed)?*

As mentioned above, in our response to the first comment of this reviewer, we have not found that less NC tension leads to more large protrusions in BCs. We have found that less NC tension results in an increase in the number of side protrusions without affecting the front and rear protrusions. In addition, our recent measurements show that the length of the front, rear and the ectopic side protrusions of laminin-depleted egg chambers is similar to that of controls (new Fig.3K). This is in contrast to what has been found in *singed* mutant egg chambers, where BCs extend protrusions of similar length from all sides of the cluster (Lamb et al. 2020). Thus, as mentioned above, we do not believe the ectopic side protrusion we found in laminin-depleted egg chambers are the same as the ones described in Aranjuez et al., 2016 and Lamb et al., 2021, and we suggest that the increase in the side protrusions may be a consequence of a less constrictive environment. We have now changed the discussion to clarify our hypothesis.

*4. About the tension property of NCs, corset property of external follicle cells should be present in apical and later basal medial region, which has also been shown in (Balaji et al., 2019) to contract apical surface of FCs to resist the expansion of internal germ cells. However, authors did laser ablation in FC junctions, but not at apical and basal medial region. Can authors explain the reason of choosing this subcellular region for laser dissection? If medial region is the major region for FC tension, how does this tension change by BM stiffness transfer inside to balance the NC tension? Can authors comment on this unclear point?*

We do not understand what the reviewer means when saying that “*medial region is the major region for FC tension”*. The work of Balaji et al. (2019) demonstrated that, contrary to naïve expectations, FCs resist the expansion of the growing germ cells by decreasing tension at adherens junctions (AJs) during stages 6 and 9, which they propose could be achieved by a shift from junctional to medial MyoII localization, by un unknown mechanism. This developmentally controlled relaxation of AJs will allow the apical surface area of FCs to smoothly adapt to that of the growing germline surface. The medial basal actomyosin contractions do not appear until late stage 9 to 10B (He et al., 2010), while we measure BC migration at early and mid-stage 9. Thus, this basal medial contraction should not affect our measurements. To measure tension at AJs, Balaji et al. used egg chambers from flies carrying a GFP knock-in allele in *shotgun* (*shg-GFP*), the gene encoding the E-cadherin protein in flies. However, we found that *shg-GFP* was detected differentially along the apical-basal axis, with high levels at the apical membrane and lower levels at the lateral AJs, which is in agreement with other studies analysing E-cadherin expression in FCs (Mallart et al. 2022). Thus, it was not clear to us where, along the apical-basal axis of the cells, Balaji et al performed their laser ablations. In addition, we wanted to use a GFP-marker that would allow us to ablate both at the apical and basal sides of FCs. Thus, we labelled FC membranes using Resille-GFP. We performed our laser ablations at the most apical side of FCs, as determined by the region of the cell closest to the germline, which is where AJs are located. However, as we did not label AJs directly, we decided to call the sites of our laser ablations, apical FC-FC contacts.

*Minor comments:*

*1. Authors are still not careful of the stage of egg chamber and BC migration for the comparison of different genotypes, such as Supple Movie 2, Supple Fig 3, Supple Fig 9. Different migratory stages can strongly affect the migration speed for sample comparison.*

In this work, we have recorded more than one hundred movies, which makes nearly impossible to film all of them throughout the whole trajectory. However, each experiment has its own control and even though we show different moments of the migratory process of BCs, depending on whether we are looking at the whole process -when using the *tj Gal4* line- or at the second half of the process -when using the *mirror Gal4* line, we always compare control and experimental egg chambers of the same stage. In addition, migration speed has always been calculated in control and experimental conditions between the same points along the trajectory.

*2. Authors argued that less tension in NCs can prevent BC migratory rotation while strong NC tension favors BC rotation. However, we noticed that in Supple movie 9 (Abi RNAi expression in NCs), BC rotation mode is even stronger than control. For rotation experiment, can authors use longer time period for this behaviour, since short-time movies (used by authors for BM stiffness changes, compared with control) might miss the rotation occurrence in some cases.*

We thank the reviewer for this comment. We have now quantified BC rotation in several control (*nosGal4*) and *nos>abiRNAi* egg chambers. We have found that while 100% of *nosGal4* rotate, rotation was hampered in 20% of *nos>abiRNAi* egg chambers analysed. This result supports our idea that less tension in NCs can prevent BC migratory rotation, which we believe contributes to increase speed. We have added this information to the text and to Sup. Fig.7.

*3. Authors mentioned twice in the text that protrusions of BCs are filopodia. However, without prominent markers of filopodia, it is not precise to judge whether protrusions are filopodia only based on their finger-liked shape. One known example is Cadherin fingers in endothelial cells which are not filopodia structure at all.*

We agree with the reviewer in that we cannot call the protrusions of BCs filopodia and in most cases we have called them protrusions. Thus, we thank the reviewer for pointed out these two occasions in which we have not done so. We have corrected this and have checked the whole text again and found no other mislabel.

*4. Line201-205 "Quantification of BC RNAi migration speed from live imaging analysis revealed that BCs from tslGFP; tj>LanB1RNAi (n=7), tslGFP; LanB1hyp (n=5) or tslGFP; tj>Coll I (n=5) egg chambers moved faster than BCs from control follicles (tslGFP; tj>Gal4, n=8) (Fig.1A-ED-H, Movie S2S1, see Materials and Methods)".*

*Should be "Quantification of BC RNAi migration speed from live imaging analysis revealed that BCs from tslGFP; tj>LanB1RNAi (n=7), tslGFP; LanB1hyp (n=5) and tslGFP; tj>vkgRNAi (n=5) egg chambers moved faster than BCs from control follicles (tslGFP; tj>Gal4, n=8) (Fig.1A-ED-H, Movie S2S1, see Materials and Methods)".*

This has been corrected.

*5. None of Supple movies had a scale bar, and Supple Movie 2/7 missed the time points.*

We have added scale bars to all Supple Movies and time points to Supple Movie 2 and 7.
